# Factors influencing antibiotic prescribing in long-term care facilities: a qualitative in-depth study

**DOI:** 10.1186/1471-2318-14-136

**Published:** 2014-12-16

**Authors:** Laura W van Buul, Jenny T van der Steen, Sarah MMM Doncker, Wilco P Achterberg, François G Schellevis, Ruth B Veenhuizen, Cees MPM Hertogh

**Affiliations:** EMGO Institute for Health and Care Research, VU University Medical Center, Van der Boechorststraat 7, 1081 BT Amsterdam, the Netherlands; Department of General Practice & Elderly Care Medicine, VU University Medical Center, Van der Boechorststraat 7, 1081 BT Amsterdam, the Netherlands; Department of Public Health and Primary Care, Leiden University Medical Center, Hippocratespad 21, 2300 RC Leiden, the Netherlands; NIVEL (Netherlands Institute for Health Services Research), Otterstraat 118 – 124, 3513 CR Utrecht, the Netherlands

**Keywords:** Antimicrobials, Drug prescribing, Nursing homes, Residential care homes

## Abstract

**Background:**

Insight into factors that influence antibiotic prescribing is crucial when developing interventions aimed at a more rational use of antibiotics. We examined factors that influence antibiotic prescribing in long-term care facilities, and present a conceptual model that integrates these factors.

**Methods:**

Semi-structured qualitative interviews were conducted with physicians (n = 13) and nursing staff (n = 13) in five nursing homes and two residential care homes in the central-west region of the Netherlands. An iterative analysis was applied to interviews with physicians to identify and categorize factors that influence antibiotic prescribing, and to integrate these into a conceptual model. This conceptual model was triangulated with the perspectives of nursing staff.

**Results:**

The analysis resulted in the identification of six categories of factors that can influence the antibiotic prescribing decision: the clinical situation, advance care plans, utilization of diagnostic resources, physicians’ perceived risks, influence of others, and influence of the environment. Each category comprises several factors that may influence the decision to prescribe or not prescribe antibiotics directly (e.g. pressure of patients’ family leading to antibiotic prescribing) or indirectly via influence on other factors (e.g. unfamiliarity with patients resulting in a higher physician perceived risk of non-treatment, in turn resulting in a higher tendency to prescribe antibiotics).

**Conclusions:**

Our interview study shows that several non-rational factors may affect antibiotic prescribing decision making in long-term care facilities, suggesting opportunities to reduce inappropriate antibiotic use. We developed a conceptual model that integrates the identified categories of influencing factors and shows the relationships between those categories. This model may be used as a practical tool in long-term care facilities to identify local factors potentially leading to inappropriate prescribing, and to subsequently intervene at the level of those factors to promote appropriate antibiotic prescribing.

**Electronic supplementary material:**

The online version of this article (doi:10.1186/1471-2318-14-136) contains supplementary material, which is available to authorized users.

## Background

Antibiotics are commonly prescribed in nursing homes and residential care homes. As much as 47% to 79% of the people residing in these facilities receives at least one course of antibiotics per year, of which a substantial part in situations where antibiotic treatment is not indicated [1]. This inappropriate antibiotic use contributes to the development of antibiotic resistance, which is also common in long-term care settings. These insights have led to awareness regarding appropriate use of antibiotics, and to several initiatives to promote rational antibiotic prescribing.

To be effective, interventions aimed at a more rational use of antibiotics should take into account the factors that impede and facilitate appropriate prescribing. Such factors may apply to the patient, the physician, the care setting, and the larger cultural and socio-economic context [2]. Factors that influence antibiotic prescribing in general practice and hospitals have been studied extensively. Examples of such factors include patients’ symptoms and results of physical examination, availability of resources, availability and awareness of evidence with regard to antibiotic treatment, diagnostic uncertainty, peer practice, patient expectations, financial interests, and physicians’ perceptions regarding antibiotic prescribing and resistance [2–14]. The diversity of these factors indicates that the antibiotic prescribing decision can be complex in these settings.

Less research has been conducted on factors that influence antibiotic prescribing in nursing homes and residential care homes. Whereas several factors identified for the general practice and hospital setting are likely to be valid – at least partly – in long-term care settings, other factors may be involved that relate to the specific characteristics of these facilities, the physicians delivering care, and the patient population. A few studies quantitatively investigated associations between antibiotic prescribing and possible determinants in long-term care facilities [15–20]. These found that prescribing decisions can be affected by, for example, the severity of illness and the ability to communicate with residents. Other studies qualitatively investigated factors that influence antibiotic prescribing for specific conditions (i.e. urinary tract infection and pneumonia), and reported that antibiotic prescribing decisions may be influenced by nursing staff, family wishes, and familiarity with the patient [21–23]. To date, factors that influence antibiotic prescribing in general have not been qualitatively explored in-depth in long-term care facilities.

Based on qualitative interviews with physicians and nursing staff, this study therefore examines factors that influence antibiotic prescribing in general in long-term care facilities in the Netherlands, where prevalence of antibiotic prescribing is high compared to ambulatory care settings and average in comparison with long-term care facilities in other European countries [24, 25]. We present a conceptual model that integrates these factors, which may guide the development and implementation of interventions aimed at rationalizing antibiotic use in long-term care facilities.

## Methods

### Study setting

The current interview study is part of a research project aimed at rationalizing antibiotic prescribing in long-term care facilities: the IMPACT study [26]. The IMPACT study was conducted in 14 long-term care facilities, of which seven were allocated to an intervention group and seven to a control group. In the interview study, which preceded implementation of interventions to improve prescribing practices, we included only facilities from the intervention group (5 nursing homes and 2 residential care homes), to avoid undue influence of participation in qualitative research activities on prescribing behavior in control group facilities.

In the Netherlands, organization of medical care differs between nursing homes and residential care homes. Nursing homes employ elderly care physicians (formerly called nursing home physicians), which is a distinct medical specialty in the Netherlands. Medical care in residential care homes is provided by general practitioners, who operate from their own practice. Interviewees were from both care settings.

All participating facilities were located in the central-west region of the Netherlands. A sample of 13 out of approximately 30 physicians was purposefully selected by the researchers to reflect variation in sex, age, years of professional experience, and professional specialism. One of the 13 initially selected physicians was not able to participate in an interview due to time constraints, and another physician was selected instead. The physicians in this final sample all provided written consent to participate in the interviews. A sample of 13 nursing staff members was additionally selected by researchers with the help of a location manager, a physician, or a medical secretary, similarly pursuing variation. These participants provided consent in person prior to the start of the interviews.

### Data collection

A team of researchers (LB, JS, SD, FS, CH) developed two topic lists (Additional file 1), one for physicians and one for nursing staff, based on field experience of the project team, relevant literature on factors associated with drug prescribing, and a literature-based conceptual model developed by Zimmerman et al [27]. Both topic lists aimed at exploring perceptions and motivations with regard to three themes: infectious diseases, antibiotic prescribing, and antibiotic resistance. For the theme ‘antibiotic prescribing’, respondents were asked to describe two recent cases: one in which antibiotics were prescribed and one in which antibiotics were not prescribed. The topic list was used to raise follow up questions to determine factors influencing prescribing decisions.

One semi-structured interview per respondent was conducted by trained interviewers (LB and SD). To achieve concordance, the interviewers conducted the first two interviews together. All interviews were tape-recorded and transcribed in full, and we removed any information from which the particular respondent or long-term care facility could be identified.

### Data analysis

We started the analysis with the recent cases that were described by physicians, as these constituted the basis of the interviews. These case descriptions were studied by two researchers (LB and SD) to identify and categorize factors that influence antibiotic prescribing decisions. The resulting categories were regarded as basic considerations for treatment decisions (i.e. they are generally considered in treatment decisions), and were therefore considered the core of a conceptual model. An iterative analysis was applied to further elaborate this conceptual model. Hereby, the remaining material of the physician interviews – which contained descriptions of other practice situations with regard to antibiotic prescribing decisions – was studied in a stepwise fashion: 1) fragments of the material were labelled according to their content (open coding), 2) relationships were sought between the coded fragments (axial coding), and 3) the related coded fragments were categorized (selective coding) and added to the conceptual model.

Open coding was conducted by two researchers (LB and SD), who independently coded transcripts of 3 physician interviews, and developed a separate code list. These code lists were compared, discussed, and combined into a collective code list. The 3 previously coded transcripts and the remaining 10 transcripts were (re)coded by each researcher according to the collective code list. After each third coded transcript, the researchers compared and discussed the transcripts and – where necessary – codes were added or adjusted according to reached consensus. Coding of the last few transcripts yielded no new codes, which indicates data saturation. Axial and selective coding was conducted by one of the researchers (LB), and discussed with the other researcher (SD). The qualitative data analysis software program Atlas.ti, version 6 (ATLAS.ti Scientific Software Development GmbH, Berlin, Germany) was used to process the coded transcripts.

Since physicians are responsible for the prescribing decision, the physician interviews were used for the initial development of the conceptual model. Subsequently, this model was triangulated with perspectives derived from the 13 coded interviews with nursing staff. The coding procedure of these interviews was identical to and independent of the procedure of the physician interviews. The information retrieved from the interviews with nursing staff was used to support and enhance the understanding of antibiotic prescribing decisions made by physicians. In addition, the conceptual model was studied by all members of the study team and adjustments to the model were made upon critical discussion of the analytic steps and interpretation of the results.

### Ethical approval

The IMPACT study was approved by the Medical Ethics Review Committee of the VU University Medical Center (Amsterdam, the Netherlands).

## Results

**Figure 1:**
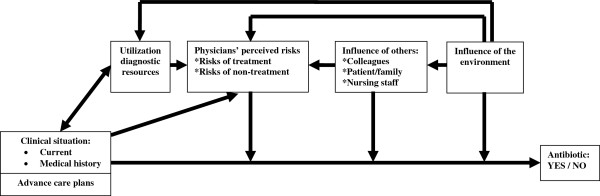
**Conceptual model of factors that influence antibiotic prescribing in nursing homes and residential care homes in the Netherlands.** The model shows that the clinical situation and advance care plans constitute the basis of the antibiotic prescribing decision. The other four categories can exert a direct influence on this prescribing decision, or an indirect influence via other categories. The *clinical situation* can influence the *use of diagnostic resources* (e.g. no X-ray when a patient is severely ill) and vice versa (e.g. less information about the clinical situation when no diagnostic resources are used). The use of diagnostic resources can also be influenced by environmental factors (e.g. availability of on-site diagnostic resources). *Physicians’ perceived risks* can be influenced by the clinical situation (e.g. higher perceived risk of non-treatment if a patient is severely ill), the use of diagnostic resources (e.g. more uncertainty if no diagnostic resources are used), others (e.g. pressure from patients), and the environment (e.g. different risk perceptions when on call). *The influence of others* can be affected by the environment (e.g. the influence of nursing staff may differ when a consultation is by telephone compared to a physical consultation).

Table 1 shows the demographic characteristics of the interviewed physicians and nursing staff; there was substantial variation in age (range: 24 – 61) and years of professional experience (range: 0 – 36). The duration of the interviews varied from 19 minutes to 53 minutes, with a mean of 34 minutes overall (physicians: 39 minutes, nursing staff: 30 minutes).The analysis of recent cases that were described by physicians led to the identification of two core categories of factors that influence the antibiotic prescribing decision: the clinical situation, and advance care plans. These categories were also derived from the analysis of other practice situations that physicians described with regard to antibiotic prescribing. The latter analysis additionally resulted in the identification of the following categories: utilization of diagnostic resources, physicians’ perceived risks, influence of others, and influence of the environment. Figure 1 shows our conceptual model that integrates these categories and demonstrates how they are interrelated. Interviews with nursing staff supported the identified categories and added no new information to the model. The categories of factors that were identified as influencing the antibiotic prescribing decision are described in more detail below.

**Table 1:** **Demographics of the interviewed physicians and nursing staff**

Demographic		Physicians (n = 13)	Nursing staff (n = 13)	Overall (n = 26)
**Sex**	Male	4	1	5
	Female	9	12	21
**Age (yr)**	Mean (range)	45 (25–60)	45 (24–61)	45 (24–61)
**Years of professional experience**	Mean (range)	15 (0–36)	17 (0–32)	16 (0–36)
**Type of facility**	Nursing home	10	9	19
	Residential care home	3	4	7
**Facility location**	Urban area	8	7	15
	Rural area	5	6	11
**Professional specialism**	Nursing home	Elderly care physician (7)	Nurse^*^ (4)	-
		Elderly care physician in training (1)		
		Junior doctor (1)	Nurse assistant^*^ (5)	
		Physician assistant (1)		
	Residential care home	General practitioner (3)	Nurse assistant^*^ (4)	

* United States equivalents: nurse = registered nurse, nurse assistant (levels 2, 3 and 4) = licensed practical nurse (level 4) or nurse aid (levels 2 and 3).

### Clinical situation

Both the current clinical situation and the patients’ medical history appeared to play a crucial role in the decision to prescribe or not prescribe antibiotics. Table 2 shows considerations with regard to the clinical situation that affect the prescribing decision for urinary tract infection, respiratory tract infection, and skin infection. Two situations were described in which the clinical situation can be unclear: 1) when communication with patients is impaired, which is common in residents with dementia, and 2) when (typical) clinical signs and symptoms are absent. Such situations result in diagnostic uncertainty, which can either promote antibiotic use if uncertainty leads to prescribing, or impede antibiotic use if uncertainty leads to further observing the course of infection. According to the interviewed physicians, a reason for not prescribing antibiotics for urinary tract infection is the absence of clinical signs and symptoms despite a positive dipstick test (i.e. the presence of leukocyte esterase, nitrite, or both). Some physicians expressed dissatisfaction with nursing staff performing a dipstick test in such situations, especially when the rationale for the test was a change in urine odor or appearance. Nursing staff, on the other hand, may not always be aware of this dissatisfaction, as some respondents indicated a change in urine odor or appearance as a reason to perform a dipstick test. This is illustrated in the following quotations:

**Table 2:** **Elements of the clinical situation that result in the decision to prescribe or not prescribe antibiotics for urinary tract infections, respiratory tract infections, and skin infections**

Clinical situation	Antibiotic	Urinary tract infection	Respiratory tract infection	Skin infection
**Current**	**YES**	Signs and symptoms (or a high risk of signs and symptoms), positive dipstick test (for leukocyte esterase, nitrite, or both)/dipslide/culture, patient experiences burden, patient feels ill, hematuria, vulnerability of the patient, comorbidity, no prior antibiotic resistance	Signs and symptoms, patient feels ill, vulnerability of the patient, risk of death, comorbidity	Signs and symptoms, vulnerability of the patient
	**NO**	Absence of (relevant) signs and symptoms whether or not in combination with a positive dipstick test (for leukocyte esterase, nitrite, or both), negative dipstick test, awaiting culture results in case of no/minimal signs and symptoms, patient does not feel ill, poor prognosis, acceptance of resistant bacteria in urine	Poor prognosis, suspected viral infection, no/minimal signs and symptoms, patient does not feel (severely) ill, physical inability to take oral medication, allowing immune system of the patient to clear infection	Absence of (relevant) signs and symptoms
**Medical history**	**YES**	Positive effect of treatment for previous infection, no/limited history of infection, ineffective previous treatment	Severe course of previous infection	-
	**NO**	-	No history of infection	-

*Elderly care physician, female, 53: “The nurses call out ‘yes, the urine stinks’. And so they started dipstick testing [the urine]. And I say ‘well I am not treating urine, I am treating the patient’.”*

*Nurse, female, 53: “Sometimes the urine is checked because it is just very nasty. Very concentrated, or it smells really bad.”*

### Advance care plans

The interviews showed that advance care plans can play a central role in the decision making process in nursing homes (they were not mentioned for residential care homes). These include the documentation of considerations to guide future (non-)treatment decisions, as formulated by the physician and the patient or the patients’ family. Antibiotic treatment may be included in the advance care plan, thereby anticipating situations in which antibiotic treatment potentially prolongs life. The interviewed physicians consulted the advance care plan when a patient develops a potentially life-threatening infection such as pneumonia. They stated to not prescribe antibiotics when the overall care goal in the advance care plan was defined as comfort rather than life prolongation.

### Utilization of diagnostic resources

The interviews demonstrated that the extent to which physicians resort to diagnostic resources is limited in long-term care facilities. Consequently, physicians have less information to judge a clinical situation compared to situations in which additional diagnostic information would be available, which in turn contributes to diagnostic uncertainty. We abstracted from the interviews four explanations for not using diagnostic resources to facilitate treatment decisions. First, certain diagnostics can be too burdensome for the vulnerable long-term care population (e.g. referring a patient to the hospital for further investigation). A second explanation includes the inability to obtain a good sputum or urine sample for culture from elderly patients. In addition, logistic considerations can be involved in the decision not to use diagnostic resources. In this regard, physicians pointed to a lack of on-site diagnostic resources (e.g. C-reactive protein point-of-care test, X-ray, urine culture), difficulties to consult the laboratory outside regular visit days for collection of specimen of residents, higher workload for the physician when taking cultures, and the length of time needed to obtain laboratory culture results (i.e. approximately one week). Finally, financial considerations can also be involved, in particular related to laboratory costs of cultures.

### Physicians’ perceived risks

The interviews showed that risks perceived by physicians can influence the antibiotic prescribing decision. These can be divided into perceived risks of treatment and perceived risks of non-treatment. With regard to perceived risks of treatment, some physicians described situations in which the risk of side effects was mentioned as one of the reasons to not prescribe antibiotics. Further, some physicians raised the risk of antibiotic resistance development, which was considered from two points of view. The first point of view was that antibiotics should not be prescribed because of the risk of antibiotic resistance, if the clinical situation does not necessarily require antibiotic treatment. The second perception was that antibiotic resistance is not an important consideration in antibiotic prescribing, as the vulnerable long-term care population has a short life-expectancy. For example:

*General practitioner, female, 38: “…if the gentleman is going to die anyway then any antibiotic resistance is not relevant. So in my mind that is something of a mitigating thing.”*

Perceived risks of non-treatment appeared to influence the antibiotic prescribing decision especially when physicians experience uncertainty, for example due to diagnostic uncertainty or unfamiliarity with the patient. We identified three situations in which perceived risks of non-treatment resulted in treating more readily with antibiotics. The first situation involves a perceived risk of adverse outcomes. For example:

*General practitioner, female, 47: “So even if I initially think well it’s only viral, but I feel there is a very substantial risk of a superimposed infection in case they have a respiratory infection, then I am just very quick [to prescribe antibiotics].”*

The second situation involves a perceived sense of alarm (i.e. a “gut feeling”). For example:

*Elderly care physician, female, 36: “… if I am not completely sure and I simply don’t trust the situation, then I will [prescribe antibiotics]. In that case I think well, better safe than sorry.”*

The third situation involves a perceived risk of not fulfilling the patients’ expectations. The quotation below shows that the physician perceives that the patient expects her to “do something,” which she interpreted as the prescription of an antibiotic:

*Elderly care physician in training, female, 25: “If I don’t take action it looks like I don’t want to help the patient, but perhaps I already know, well is it going to work at all?”*

### Influence of others

Physicians described several situations that showed influence of others on the prescribing decision. These can be colleagues, the patient, the patients’ family, and nursing staff. Some situations showed that physicians may be more susceptible to the opinion or wish of others in uncertain situations. Vice versa, the opinion or wish of others may also affect the degree of uncertainty experienced by physicians.

Three situations in which colleagues influenced the prescribing decision were described: 1) following the advice of a colleague when in doubt about whether to treat with antibiotics or not, 2) an agreement to treat patients according to the habits of a colleague when covering for this colleague, 3) adaptation to prescribing habits of peers. The latter is illustrated by the following quote:

*Physician assistant, male, 51: “That is during the weekend […] and then almost everybody prescribes Augmentin [i.e. amoxicillin-clavulanate]. That’s why. That was my motivation too.”*

Physicians and nursing staff described several situations in which patients or the patients’ family expressed their wish with regard to the treatment of an infection. Based on these descriptions, we identified three scenarios of how physicians handle these situations: 1) physician complies with a wish not to treat, 2) physician complies with a wish to treat, and 3) physician does not comply with a wish to treat. These are described and illustrated with relevant quotations in Table 3.

**Table 3:** **Scenarios of how physicians handle situations in which patients or the patients’ family express their opinion or wish regarding the treatment of an infection**

Scenario	Description of situation	Relevant quotations
**Physician COMPLIES with patients’/family’s WISH NOT TO TREAT**	Physicians indicate to not prescribe antibiotics when the patient or his/her family does not want life-prolonging antibiotic treatment (often recorded in advance care plans).	*Junior doctor, female, 30: “…if the family really decides not to do it [treat with antibiotics], then they accept the risk that he [the patient] will die as a result of it. And who am I to say well I am going to give antibiotics anyway. At that point that is not my role. Then I just have to accept what they want.”*
**Physician COMPLIES with patients’/family’s WISH TO TREAT**	Antibiotic treatment is considered necessary by physician.	
	Antibiotic treatment is considered (partly) medically futile by physician, but:	
	• family wants to have time to deliberate with a family member that cannot be reached, in case of a poor prognosis of the patient.	*Elderly care physician in training, female, 25*: *“… then I decided in consultation with his son to start the antibiotics […] because another son was on holiday […]. And we couldn’t get a hold of him on the phone.”*
	• physician is willing to concede to the wish of family due to unfamiliarity with the patient and inability to predict the outcome.	*Junior doctor, female, 30: “… if they [the family] insist, then we should do it [prescribe antibiotics] because I don’t know the man. So it’s difficult to predict. I think it won’t make much of a difference, but still, if the family really insists, then I am quite willing to prescribe [antibiotics].”*
	• physician considers it unethically to ignore the religion-based wish of the patient/family, in case of a poor prognosis of the patient.	*General practitioner, female, 38: “…I think it is very unethical to say at a moment like that I’m sorry, but you are not getting them [antibiotics]. Even if everything in me says you’re not going to make it, this is literally the last mile, but the gentleman feels like ‘I’ve done everything, if I die now then it must be God’s will’.”*
	• a perception that scientific research showed that the outcome of a pneumonia is not much influenced by treatment with antibiotics [in case of respiratory tract infections at the end-of-life].	*Elderly care physician, male, 51: “…now we also know from scientific research that if you talk about pneumonia that the outcome […] is not really determined by whether you use an antibiotic or not. And that makes it a little easier for us to give it even when you think ‘well, if it was my mother I wouldn’t have done this’.”*
	• family should be allowed time to get used to the idea that the condition of a patient deteriorates.	*Elderly care physician, male, 48: “… I just happened to have had some patients recently of whom I thought in retrospect I just shouldn’t have done it [prescribed antibiotics]. But sometimes you do it for the family. […] In the past I used to be more principled about this, I would say look, you shouldn’t do this, and now I think well, it’s a process for them too and I do tell them [that there is not much point], but if they can’t go along with that yet then I don’t push harder.”*
	• patients on rehabilitation units are used to get antibiotics from their general practitioner and will consult this general practitioner if no antibiotic is provided.	*Nurse assistant, female, 35: “[That is because] people are a bit more articulate of course [on the rehabilitation unit]: ‘[…] I just have a urinary tract infection’. And this is treated in the home situation. So sometimes that is the reason that the physician does treat it here, sometimes […]”*
**Physician DOES NOT COMPLY with patients’/family’s WISH TO TREAT**	Antibiotic treatment is considered medically futile by physician.	*Elderly care physician, female, 53: “…and some patients […] then demand treatment. […] When I am convinced that ‘this is pointless, this is medically completely pointless’. Then I don’t do it [prescribe antibiotics].”*
	Family of a mentally competent patient wants treatment whereas the patient does not want treatment.	*Elderly care physician, female, 53: “Well it depends […], if someone is competent. And this person says ‘no’ [no antibiotics] but the family says ‘yes’ [give antibiotics], then I also say I won’t do that. Because your mother is quite clear about it.”*

The interviews showed indirect and direct influence of nurses and nurse assistants on treatment decisions of physicians. Indirect influence includes the dependence of physicians on nursing staff for information about the clinical situation of a patient: the poorer the quality of the information or the conveyance of information, the more difficult it can be for a physician to assess the clinical situation and make a treatment decision. Physicians’ opinions differed about the quality of information obtained and conveyed by nursing staff. Some mentioned that nursing staff is well-capable of recognizing signs of infection and judging when the physician should see a patient, others indicated that the quality of information and conveyance of information depends on the experience and level of education of the nursing staff member. The quality of information conveyance can also be influenced by the work schedule of nursing staff; staff that had the previous days off may not be as informed about the clinical situation of a patient as staff that personally witnessed the course of illness. Furthermore, some physicians mentioned that their treatment decision is often complicated by the omission of nursing staff to register the patients’ temperature, blood pressure, and pulse.

With regard to direct influence of nursing staff, several situations were described in which nursing staff expressed a request for antibiotic treatment. For example:

*Nurse, female, 53: “Then I sometimes call directly to say ‘there are unmistakable signs of an infection, come and prescribe antibiotics’.”*

Whereas some physicians reported not to comply with such requests in situations where they considered antibiotic treatment medically futile, others indicated that they value and comply with the opinion of nursing staff in certain situations, for example:

*Elderly care physician, female, 36: “When a nurse has serious concerns I think I would be more tempted to prescribe an antibiotic, […] Nurses are often good judges of patients because they know them much longer than I do.”*

### Influence of the environment

The interviews demonstrated that the antibiotic prescribing decision can be influenced by several environmental factors. These include the availability of evidence with regard to treatment of infections. Some physicians reported that treatment decisions are complicated by a lack of prescribing guidelines for the older population, and a lack of insight into local resistance patterns. Another environmental factor is the lack of on-site diagnostic resources, which contributes to the limited extent to which diagnostic resources are utilized. In addition, limited accessibility of information in medical files can complicate antibiotic prescribing decision making. Two other environmental factors, which are often related, are the organization of cross-covering, and familiarity with patients. Some physicians indicated that they tend to treat more readily with antibiotics when on call, due to unfamiliarity with patients:

*Elderly care physician, female, 57: “We have discussed this with the partners in our call group. That you are much quicker to give antibiotics in the weekends. Just because these patients, these families are strangers. You don’t know them very well.”*

Further, the conduction of telephone-consultations can affect the degree to which others influence treatment decisions. For example, some physicians indicated that they are more dependent on nursing staff in case of a telephone consultation. A final environmental factor that can influence antibiotic prescribing decisions is the day of the week a consultation takes place. For example:

*Elderly care physician, male, 48: “Fridays it’s always more difficult than on Mondays [to use antibiotics prudently]. […] on Fridays I think […] well, someone else is going to come in and have a look [during the weekend], he won’t be able to compare and will prescribe the antibiotics anyway, so I might as well prescribe it today. Otherwise this colleague will have to come in especially tomorrow.”*

## Discussion

Qualitative interviews with physicians and nursing staff in seven long-term care facilities in the Netherlands showed the following categories of factors that can influence antibiotic prescribing decisions: the clinical situation, advance care plans, utilization of diagnostic resources, physicians’ perceived risks, influence of others, and influence of the environment. In-depth analysis of these categories showed several factors that may result in inappropriate antibiotic prescribing decisions, such as risk avoidance (‘better safe than sorry’), adaptation to peer practice, and pressure exerted by patients, family members or nursing staff. We developed a conceptual model that integrates the categories of factors and demonstrates how they may interrelate. This model may be used as a practical tool, whereby facilities explore which local non-rational factors influence their prescribing patterns, and subsequently intervene at the level of those factors to promote appropriate prescribing.

We identified the clinical situation and advance care plans as the two core categories of factors that influence antibiotic prescribing, and these therefore constitute the basis of the conceptual model. In line with our findings, these categories were among the most important factors in a Dutch study that quantitatively investigated treatment decisions with regard to pneumonia in nursing home residents with dementia [18]. We are not aware of any other studies that investigated the role of advance care plans in the antibiotic prescribing decision making process in long-term care. Future research may further elucidate this role.

A lack of on-site diagnostic resources was previously described to result in limited utilization of diagnostic resources in long-term care facilities [22, 28–30]. Other factors that reportedly contributed to this limited utilization include the length of time needed to obtain laboratory results, and difficulties in obtaining appropriate specimens for culture, which corresponds with our findings [22, 30]. In addition, another Dutch study described limited use of procedures such as x-ray examination in the vulnerable nursing home population, which indicates that the burden of diagnostic measures for residents can be a reason not to use these [19]. Limited utilization of diagnostic resources contributes to diagnostic uncertainty. We found that other contributors to diagnostic uncertainty include impaired communication, and absence of clinical signs and symptoms, which is supported by other long-term care studies [21, 29, 30].

Our finding that nursing staff, patients, and family can influence the antibiotic prescribing decision corresponds with previous long-term care studies [16, 18, 20–23]. We found that most of the situations in which physicians complied with family wishes to prescribe antibiotics involved end-of-life situations. Other situations in which physicians took the opinion of others into account include uncertain situations, which is supported by a Dutch study on treatment decisions for nursing home residents with dementia who develop pneumonia [18]. The influence of patients and family members on antibiotic prescribing decisions can differ between countries. For example, it was found that prescribing decisions of physicians in the United States were more strongly guided by family wishes than were those of their Dutch counterparts [18, 23].

Other previously-reported factors that can influence prescribing decisions in long-term care include physicians being more inclined to prescribe antibiotics just before the weekend [22], and physician familiarity with the patient or the patients’ family [20]. In our study, a lack of familiarity with the patient or the patients’ family appeared to play a role particularly when a physician was cross-covering, and less so during regular work hours. This is likely due to the organization of nursing home care in the Netherlands; elderly care physicians are employed by the nursing home, and as their main site of practice, this facilitates the development of a relationship between the physician and their patients and patient’s family, and ensures that the physician is well-aware of their treatment preferences [31]. In countries where physician practice in nursing homes is often organized differently, such as in the United States, unfamiliarity with nursing homes residents is common [20, 23]. In line with our findings, unfamiliarity with patients can promote antibiotic prescribing due to fears of adverse outcomes [21].

Some of the factors we identified in the present study have, to our knowledge, not been described before for the long-term care population, but have been reported in the general practice or hospital setting. These include a lack of insight into local resistance patters and a lack of awareness of prescribing guidelines [3, 4, 9, 12]. In addition, prescribing habits of peers, also referred to as “prescribing etiquette”, was reported as an important factor in the antibiotic prescribing decision in hospitals and general practice [7, 10, 13]. Other factors previously-reported in these settings are related to physicians’ perceived risks. In line with our findings, the risk of antibiotic resistance development influenced the prescribing of a minority of physicians in two qualitative general practice studies [7, 12]. Furthermore, the risk of adverse outcomes in case of non-treatment, and a perceived duty towards the patient were previously reported to influence prescribing decisions [2–4, 7–10].

Two factors that were reported to influence antibiotic prescribing in other settings were not found in the present study. We did not identify disagreement or distrust with regard to existing evidence, [7, 10, 11] which may be explained by the opinion of interviewed physicians that there is not enough evidence regarding treatment of infections in long-term care. Second, the interviews did not show evidence of a direct influence of financial considerations on antibiotic prescribing [3, 10]. However, regarding utilization of diagnostic resources, financial considerations were mentioned in the present study, and so may affect antibiotic prescribing indirectly.

A strength of the current study is that the antibiotic prescribing process was investigated from the perspective of both physicians and nursing staff. As these parties collaborate and depend on each other in daily practice, we believe that our findings provide a good insight into factors that influence antibiotic prescribing in long-term care facilities. An additional strength is that we focused on recent case descriptions in the interviews, and subsequently explored other practice situations. This approach facilitates a realistic representation of daily practice with regard to antibiotic prescribing decisions.

A limitation of the study, inherent to qualitative research, is that no assumptions can be made regarding the weight that each identified factor adds to the prescribing decision. Future quantitative research is needed to elucidate the contribution of each factor to the antibiotic prescribing decision. Another limitation is that our study design did not allow for checking data saturation at the time of data collection. However, no new codes appeared when coding the last few interviews, which supports that a sufficient amount of data was collected for drawing conclusions on this topic.

A proper analysis of relevant factors that influence antibiotic prescribing is crucial for the development of an antibiotic prescribing improvement program [3]. Several studies show that interventions that target factors that impede appropriate antibiotic prescribing are likely to be more effective [32–34]. The conceptual model presented in this study may be used as a practical tool, whereby facilities explore, for each category in the model, which factors influence local antibiotic prescribing, and identify which of these are inappropriate. Subsequently, they can intervene at the level of inappropriate factors to promote rational antibiotic prescribing. For example, if pressure exerted by patients is identified as a factor leading to inappropriate prescribing, interventions such as patient education could be implemented to address this factor. Factors resulting in inappropriate prescribing may differ between facilities and nations. For instance, influence of nursing staff on the prescribing decision may be more important in facilities where – unlike in the Netherlands – no on-site physicians are present, and where many consultations are conducted by telephone. In addition, the extent to which diagnostic resources are used may differ between facilities, with some facilities having better access to such resources than others. Whereas the importance of each factor in decision making may differ between facilities and nations, we believe that our model in general is likely to be widely applicable as many of the factors that we incorporated in the model have been reported in a variety of settings and countries. In addition, it shows overlap with a literature-based prescribing decision model developed in a long-term care study conducted in the United States, [27] as well as with elements of a more general model for physician adherence to clinical practice guidelines [35].

## Conclusions

Our qualitative study shows a variety of factors that influence antibiotic prescribing in long-term care facilities, of which several may lead to inappropriate antibiotic use. Some of these factors have not been previously reported for the long-term care setting, but have been described in studies in the general practice and hospital setting, indicating that several factors involved in these settings also apply to the long-term care setting. We developed a conceptual model that shows the relationships between the identified factors. This model may be used as a practical tool to identify local factors potentially leading to inappropriate prescribing, to guide the development of antibiotic prescribing improvement programs that target these factors.

## Electronic supplementary material

Additional file 1: **Topic lists for physician (A) and nursing staff (B) interviews.** Description of data = Topic lists used for qualitative interviews with physicians (A) and nursing staff (B), on infectious diseases, antibiotic prescribing, and antibiotic resistance. (PDF 41 KB)
