# Supplementary material for: Factors influencing antibiotic prescribing in long-term care facilities: a qualitative in-depth study
Source: BMC Geriatr. 2014 Dec 16;14:136. doi: 10.1186/1471-2318-14-136 (PMC4289541; doi:10.1186/1471-2318-14-136)
Supplement: Supplementary file 1 — Additional file 1: Topic lists for physician (A) and nursing staff (B) interviews. Description of data = Topic lists used for qualitative interviews with physicians (A) and nursing staff (B), on infectious diseases, antibiotic prescribing, and antibiotic resistance. (PDF 41 KB) [file 12877_2014_1071_MOESM1_ESM.pdf]

A) Topic list for physician interviews

Instruction: ***bold-printed topics*** *represent the essential elements to be covered in the interview, the remaining questions can be used to raise follow-up questions.*
[Instructions to interviewer are printed in italics]

Infectious diseases

- Can you tell me something about the **occurrence of infectious diseases** in residents of this facility?

> - How often do they occur (in comparison with other diseases)?
> - Which types of infectious diseases occur most often?
> - According to you, how does the occurrence of infectious diseases in this facility compare to the occurrence of infectious diseases in other healthcare settings (other nursing homes / residential care facilities, hospitals, general practices, etc.)

Antibiotics

- Can you describe the **most recent case in which you prescribed antibiotics**?

Listen carefully to which of the below mentioned topics are raised, and relate to these in follow-up questions accordingly. Note: not all questions (those not bolded) need to be covered!

- What aspects of this case resulted in your decision to prescribe antibiotics? In other words, **which considerations did you make** prior to your decision to prescribe antibiotics?

> *In case the clinical presentation/status of the patient is considered in the prescribing decision:*
> *(E.g. signs and symptoms, additional diagnostic information, clinical history)*
> - Was the clinical presentation clear?
>   - Is the clinical presentation often (also) clear in other cases?
>   - Are there any patient groups in which the clinical presentation is often less clear?
> - Do you find proper diagnosing difficult if the clinical presentation is ambiguous or not clear?
>
> In the described case, **what information did you need from the nursing staff**?
> - Did you indeed get this information?
> - How do you feel about the quality of the information in this case? Is this in line with your opinion more generally?
>   - Are there any differences in the quality of information if you ask a patient for his/her symptoms yourself compared to if you obtain this information via the nursing staff?

- In your opinion, is nursing staff capable of adequately assessing signs and symptoms of infections?
- In your opinion, does nursing staff adequately report signs and symptoms of infections to the physicians?
- Does the provision of information by telephone affect the quality of the information?

*In case of **influence of the patient, family and/or nursing staff** on the prescribing decision:*
(if this did not appear from the case description, inquire about any occurrence of influence of the patient, family and/or nursing staff more generally, and ask for an example if applicable).

- Which preferences were expressed by patients, family and/or nursing staff? Are such preferences expressed more frequently?
- To what extent did these preferences concur with the treatment you would propose? Do they (also) concur in other cases?
- Do you experience it as ‘difficult’ to handle the preferences of patients, family and/or nursing staff? Why do/don’t you experience this as ‘difficult’? In which situations in particular?
- Does the duration or quality of the physician-patient relationship affect the way preferences of patients and family are dealt with? And how so?

In case other factors appeared to be involved in the prescribing decision:
(E.g. the risk of development of antibiotic resistance, organizational factors (time pressure, staff shortage, staff turnover, presence/availability of diagnostic resources, financial considerations).
Thoroughly question how and why these factors were considered!

- In retrospect, do you feel that **prescribing antibiotics** was the right decision in this case?

- Can you explain this? Why do you feel this was (not) a good decision?
  In case treatment effectiveness supported the feeling that prescribing antibiotics was the right decision:
- Based on what did you conclude that the treatment was effective?

- What is your understanding of a ‘good’ prescribing pattern?
  (Based on evidence, a formulary, routine, experience, observed effectiveness, etc.)
- **Generally spoken** (not specifically for this case), in retrospect, do you consider your decision to prescribe antibiotics as the right/a good decision or not the right/a good decision?
  - Can you explain this?
- Can you describe a situation in which you felt, in retrospect, that your decision to prescribe antibiotics was not a good decision? Or that you were in doubt about whether it was a good decision or not?

- Do you believe that there are opportunities to improve antibiotic prescribing by physicians (including yourself)?
  - Can you give examples of such opportunities?
  - How could this be achieved?
  - Do you believe physicians are open to such opportunities?

- Why did you **select the specific antibiotic agent** prescribed in the described case?
  (E.g. formulary, patient allergies, patients' renal function, prior antibiotic resistance)

> - Was this choice based on empirical grounds, or did you have any information about the infective agent (culture result) at the time of prescribing?
> - Is your prescribing often empirically / based on information about the infective agent in other situations as well?
>
> - When do you decide to take a culture?
> - What prevents you from taking cultures?

- Is … [infection type case] an **infection type for which you often prescribe antibiotics**?

> - Why is / isn't this an infection type for which you often prescribe antibiotics?
> - For which infection types do you also frequently / more frequently prescribe antibiotics?
> - **In general**, are antibiotics frequently prescribed for the residents of this facility?
>   - According to you, how does the occurrence of antibiotics prescribing in this facility compare to the occurrence of antibiotic prescribing in other healthcare settings (other nursing homes / residential care facilities, hospitals, general practices, etc.)

- So far, we discussed situations in which antibiotics were prescribed. Can you also describe the **most recent case [with an infection] in which you did not prescribe antibiotics**?

> - Why did you decide not to prescribe antibiotics?
> - Can you describe other situations in which you do not prescribe antibiotics?
> - Are there any situations in which you find it difficult not to prescribe antibiotics? Can you describe these situations? Why do you experience it as difficult to not prescribe antibiotics in these situations?
>   *(E.g.* ***pressure of family*** *(see also previous page) , risk of negative outcome of infection)*

<u>Antibiotic resistance</u>

- Can you tell me something about the **occurrence of antibiotic resistance in this facility**?

> - How often does it occur?
> - Do you believe there is an increase in antibiotic resistance? What are the causes?
> - According to you, how does the occurrence of antibiotic resistance in nursing homes / residential care facilities compare to the occurrence in hospitals or the community?
> - In your opinion, how large is the resistance problem in nursing homes / residential care facilities? And how large in the Netherlands in general?
> - Do you believe that you are well-aware of the developments with regard to antibiotic resistance? If not, why not?

- Do you, as a physician, **experience a personal responsibility for the emergence of antibiotic resistance** in the facility? And in society in general?

• Who is/are also/more responsible for the emergence of antibiotic resistance?

B) Topic list for interviews with nursing staff

Instruction: ***bold-printed topics*** *represent the essential elements to be covered in the interview, the remaining questions can be used to raise follow-up questions.*
[Instructions to interviewer are printed in italics]

<u>Infectious diseases</u>

- Can you tell me something about the **occurrence of infectious diseases** in residents of this facility?

> - Do they occur often (in comparison with other diseases)?
> - Which types of infectious diseases occur most often?
> - According to you, how does the occurrence of infectious diseases in this facility compare to the occurrence of infectious diseases in other healthcare settings (other nursing homes / residential care facilities, hospitals, general practices, etc.)

- **How do you recognize an infection** in a resident**?**

> - What is specific for a urinary tract infection?
>   - When do you decide to **perform a dipstick test**?
> - What is specific for a respiratory tract infection?
> - What is specific for a skin or wound infection?
>
> - Do you believe that you (and your colleagues) are well-capable of assessing signs and symptoms of infections? Why (not)?

- Can you describe **when you report signs and symptoms of infection to a physician**?

> - **When is it important to consult a physician** in case of signs and symptoms of an infection**?**
>   - Do you find it difficult to determine when a physician should be consulted for a resident?
>   - Can you describe an example of situations in which a physician is sometimes consulted too soon? And an example in which a physician is not consulted soon enough?
> - Do you believe that you are **well-capable of reporting information about signs and symptoms of infections** to a physician?
>   - Does the information that you provide help the physician in making decisions regarding the treatment of the infection?
>   - Do you feel that the physician finds the information that he/she receives important?
>   - Do you believe that at times you could provide more or better information to the physician? Or, by contrast, provide less information?
>   - How do you report the information to the physician? In person, by telephone, via written communication?

- Can you describe the **most recent situation in which you consulted a physician for a possible infection** in a resident?

> - From which type of infection did the resident suffer?
> - How did you recognize this infection?
> - How did you inform the physician?
> - How did the physician respond?
>
> - Did you expect the physician to initiate a specific treatment?
> - Did the physician act according to your expectation?
> - Can you give examples of situations in which the physician did and did not act according to what you had expected?

<u>Antibiotics</u>

- Can you tell me something about the **use of antibiotics in this facility**?

> - Are antibiotic frequently / infrequently prescribed for the residents?
> - Do you think that this pattern differs from other nursing homes / residential care homes, or other health care settings such as hospitals or general practices?
> - For which type of infection are antibiotics prescribed most frequently?

- If a resident with an infection receives antibiotics, **to what extent does this alleviate the signs and symptoms**?

> - Can you give an example of a situation in which signs and symptoms were relieved, and of a situation in which they not?
> - In the situation in which signs and symptoms were not relieved; according to you, why not?
> - In retrospect, do you often believe that the decision of a physician to start antibiotics was a good decision? Why (not)?
>   - When do you believe a physician should start antibiotic treatment? (E.g. when you feel the patient needs antibiotics, when indicated by guidelines, when antibiotic treatment is effective, etc.)

- Could physicians improve their antibiotic prescribing in any manner?

> - Can you give some examples?
> - How could this be achieved?
> - Do you believe physicians are open to such activities?

- Do physicians, at times, **not prescribe an antibiotic, while you believe it would be better if he/she did so**?
  (possible overlap with the last question of the section 'infectious diseases')

  - Can you give an example?
  - Do you then let the physician know that you believe it would be better to prescribe antibiotics? And how do you let him/her know?
  - Do you find it difficult to express your disagreement with the physician's treatment decision?

- **Can you think of any negative consequences of the use of antibiotics?**
  (E.g. with regard to side effects, development of antibiotic resistance, and costs)

  Thoroughly question why the mentioned consequences are a negative effect of antibiotic use!

<u>Antibiotic resistance</u>

- When antibiotics are used frequently, antibiotic resistance can occur. **Can you explain what this means, according to you**?
  (If they do not know: antibiotic resistance means that bacteria that cause infections are not susceptible anymore for specific types of antibiotics, and consequently, patients with these infections cannot be treated anymore with these types of antibiotics).
  (possible overlap with the previous question)

- Can you tell me something about the **occurrence of antibiotic resistance in this facility**?

  - Does it occur often?
  - Do you believe there is an increase in antibiotic resistance?
  - According to you, how does the occurrence of antibiotic resistance in nursing homes / residential care facilities compare to the occurrence in hospitals or the community?
  - In your opinion, how large is the resistance problem in nursing homes / residential care facilities? And how large in the Netherlands in general?
